# “At least i am doing something”: a health belief model-guided qualitative study of nutritional supplement use among cancer patients during treatment

**DOI:** 10.3389/fnut.2026.1908173

**Published:** 2026-08-04

**Authors:** Kejimu Sunzi, Xiaoyu Fan, Jiahao Wei, Qi Huang, Yao Zeng, Wen Zhou, Cheng Lei

**Affiliations:** 1 Department of Nursing, Deyang People's Hospital, Deyang, China; 2 Department of General Surgery, Zigong Fourth People's Hospital, Zigong, China; 3 School of Nursing, Zhejiang Chinese Medical University, Hangzhou, China; 4 Department of Nursing, The Second Affiliated Hospital of Chongqing Medical University, Chongqing, China; 5 Department of Thoracic Surgery, Sichuan Cancer Center, Sichuan Clinical Research Center for Cancer, Sichuan Cancer Hospital and Institute, The Affiliated Cancer Hospital of University of Electronic Science and Technology of China, Chengdu, China

**Keywords:** cancer, decision-making, health belief model, nutritional supplements, oncology, patient experience, qualitative research

## Abstract

**Background:**

Nutritional supplement use is widespread among people receiving cancer treatment, yet the motivations, belief structures, and lived experiences that shape these decisions are incompletely understood. This study applied the Health Belief Model (HBM) as a guiding theoretical framework to explore how and why adult cancer patients decide to use nutritional supplements during treatment.

**Methods:**

A qualitative descriptive design was used. Twenty-five adults with pathologically confirmed malignant diagnoses participated in semi-structured interviews framed around the six core constructs of the HBM. Recruitment took place between August 2025 and January 2026. Data were analyzed using a hybrid deductive and inductive thematic approach. Reporting followed the 32-item Consolidated Criteria for Reporting Qualitative Research (COREQ) checklist.

**Results:**

Four core themes and 10 sub-themes were identified: (1) seeking control in an uncertain world; (2) navigating a fragmented information landscape; (3) the body as a battleground, supplements as a tool for resilience; and (4) silent conversations, disclosure, distrust, and the patient–provider relationship. These themes mapped closely onto HBM constructs including perceived benefits, perceived barriers, cues to action, and self-efficacy. Participants described supplement use as a rational, experience-driven response to the physical and psychological pressures of cancer and its treatment, rather than as a rejection of conventional medicine.

**Conclusions:**

The HBM provides a coherent lens for understanding supplement use behavior in cancer patients. The findings point to a substantial unmet need for non-judgmental, individualized nutritional guidance within oncology care. They support a shift toward collaborative, harm-reduction-oriented communication and better integration of qualified clinical nutrition professionals, such as registered dietitians or formally trained clinical nutritionists with oncology experience, into routine cancer care.

## 1 Introduction

Nutritional supplement use is notably prevalent among people with cancer. Studies consistently report that between 68% and 73% of cancer survivors use vitamins or mineral supplements, compared with approximately 50% of the general adult population (1). Rates tend to be higher in specific diagnostic groups, particularly breast cancer, and many patients begin supplements or broaden the range they use following diagnosis (2). These patterns reflect an active desire among patients to participate in their own care during treatment, and they sustain a multi-billion-dollar industry worldwide (2).

This widespread use sits in tension with the caution expressed in mainstream oncology guidelines. Recommendations from organizations such as the American Cancer Society and the World Cancer Research Fund generally advise patients to obtain nutrients from food and express reservations about routine supplement use during active treatment (3, 4). The caution stems partly from the absence of large, high-quality randomized controlled trials demonstrating that supplements improve cancer outcomes (5), and partly from concerns about harm. Certain antioxidants, including high-dose vitamins C and E, may reduce the efficacy of chemotherapy and radiotherapy by interfering with the oxidative mechanisms that these treatments use to destroy tumor cells (6, 7). Herbal preparations such as St John's wort have been shown to alter the metabolism of specific chemotherapy agents, potentially reducing their effectiveness or increasing toxicity (8).

Between patients' active use of supplements and clinicians' caution sits a substantial communication gap. A survey revealed that among 1,479 outpatient visits, only 357 cases (accounting for 24.2%) involved discussions between physicians and patients regarding dietary supplements (9). This information asymmetry carries real patient safety implications: clinicians making treatment decisions without complete information face an unquantified risk of drug-nutrient interactions. From the patients' perspective, the gap often originates in an unmet information need. Patients commonly report wanting guidance on nutrition and supplements but feeling that they receive little individualized advice from their oncology teams (10). International evidence suggests that nondisclosure is also common outside China. In a US national sample, common reasons for nondisclosure included clinicians not asking and patients believing physicians did not need to know (11). Integrative oncology clinic data further indicate that supplement regimens can be complex and may require professional review, with discontinuation recommended for some patients because of interaction, toxicity, or excessive-dose concerns (12). Faced with this vacuum, many turn to the internet, social media, family and friends, and other lay sources, where information quality is highly variable and frequently contradictory (13).

Although quantitative research has described who uses which supplements, the deeper motivations, decision-making processes, and lived experiences underlying these behaviors are less well characterized. To examine these systematically, the present study applied the Health Belief Model (HBM) as its guiding theoretical framework (14, 15). The HBM proposes that health-related behavior is shaped by an individual's perception of disease threat, specifically perceived susceptibility and perceived severity, alongside a weighing of the perceived benefits and perceived barriers associated with taking action. Behavior is further triggered by internal or external cues to action and modulated by self-efficacy, the individual's confidence in their ability to perform the behavior (16, 17). In the context of cancer, a diagnosis serves as a powerful cue to action and heightens perceived susceptibility and severity, prompting patients to weigh the benefits and barriers of supplement use against their sense of self-efficacy (15).

This study therefore aimed to use in-depth semi-structured interviews, guided by the HBM, to explore the experiences of adult cancer patients regarding their use of nutritional supplements during treatment. The focus was on their motivations, decision-making processes, information-seeking behaviors, and interactions with their healthcare teams.

## 2 Methods

### 2.1 Study design

A qualitative descriptive design was used, with the HBM as the overarching theoretical framework guiding study design, data collection, and analysis (18). This approach supports in-depth exploration of subjective experience while the theoretical framework structures and contextualizes that exploration (18). The aim was not to generate statistically generalisable findings but to provide a theoretically grounded, contextually rich understanding of supplement use in this population. Reporting followed the 32-item COREQ checklist (19).

### 2.2 Sampling and recruitment

Purposive sampling was used to recruit participants who could speak to the research questions with depth and breadth (20). Recruitment continued until thematic saturation was reached, the point at which new interviews ceased to generate new concepts or themes (20, 21). Saturation was provisionally identified after 23 interviews and confirmed after two additional interviews, yielding a final sample of 25 participants.

Eligible participants were adults aged 18 or older with a pathologically confirmed malignant diagnosis who were currently undergoing or had recently completed (within 12 months) active anti-cancer treatment, including surgery, chemotherapy, radiotherapy, targeted therapy, or immunotherapy. Participants also had to report regular use of at least one nutritional supplement following diagnosis; supplements were defined as vitamins, minerals, herbal preparations, amino acids, probiotics, or other non-prescription products taken in addition to the prescribed treatment regimen. Participants were excluded if they were unable to provide informed consent, were unable to complete a telephone or video interview because of severe illness or cognitive/communication impairment, or used only clinician-prescribed oral nutritional products rather than self-selected non-prescription supplements.

To capture a range of experiences, sampling sought diversity in age, sex, cancer type (including respiratory, gastrointestinal, breast, and urogenital cancers), and disease stage. Education and income were not prespecified sampling strata and were not collected systematically enough to support analysis. Participants were recruited through oncology clinics, cancer support communities, and social media platforms. Recruitment took place between August 2025 and January 2026.

### 2.3 Data collection

Data were collected through in-depth semi-structured interviews, all conducted by two members of the research team (KS and XF), female registered nurses with several years of clinical care experience and formal training in qualitative interviewing. KS led the interview process, while XF was responsible for supplementing any potentially omitted information during recording. Interviews were conducted by telephone or video call, lasted between 35 and 75 min (mean 46 min), and were audio-recorded with participants' consent. The interview guide was structured around the six core constructs of the HBM: perceived susceptibility, perceived severity, perceived benefits, perceived barriers, cues to action, and self-efficacy (16). This design ensured systematic coverage of all theoretically relevant domains while preserving the flexibility to probe unexpected themes. The full interview guide is provided as (supplementary Appendix A). All recordings were transcribed verbatim within 24 h of the interview.

### 2.4 Data analysis

A hybrid deductive and inductive thematic approach was used (22, 23). Deductive coding preceded inductive coding. An initial coding framework derived from the six HBM constructs was applied independently by two members of the research team (KS and XF) to all transcripts. Segments that did not map onto HBM constructs were subsequently coded inductively, allowing patterns emerging from the data to be captured without forcing them into the pre-existing framework (22). Coded material was then organized into higher-order themes and sub-themes through an iterative process of comparison and grouping. Where the two coders disagreed, a third team member (CL) was consulted.

Trustworthiness was maintained through investigator triangulation (independent coding by KS and XF, with CL as arbiter), peer debriefing (regular discussion of emerging findings with a qualitative research specialist outside the team), and a detailed audit trail documenting all analytical decisions from raw data to final themes (24).

### 2.5 Ethical considerations

This study adhered to the Declaration of Helsinki and was approved by the Ethics Committee of Deyang People's Hospital (Approval No.2025-04-150-K02). Before data collection, all participants received written information about the study's purpose, procedures, potential risks, and anticipated benefits, and provided written informed consent. The consent process emphasized voluntary participation and the right to withdraw at any time without consequence. All identifying information was anonymised; audio recordings and transcripts were stored on encrypted local devices using participant codes, accessible only to the core research team.

## 3 Results

### 3.1 Characteristics of participants

Table 1 presents characteristics of the 25 participants (12 women, 13 men; mean age 52.3 years, range 34–71 years). Lung cancer was the most common diagnosis (*n* = 8, 32%), followed by breast cancer (*n* = 5, 20%), gastric cancer (*n* = 4, 16%), colorectal cancer (*n* = 3, 12%), and others. Disease stage ranged from stage I to stage IV. Education and income are not reported because these variables were not collected systematically across all interviews.

**Table 1:** Sociodemographic and clinical characteristics of participants (*N* = 25).

ID	Age	Sex	Cancer type	Stage at diagnosis	Time since diagnosis (y)
P01	45	F	Breast	II	0.5
P02	68	M	Lung	IV	1.7
P03	52	M	Colorectal	III	1.3
P04	39	F	Cervical	II	3.5
P05	71	M	Prostate	III	2.5
P06	58	F	Ovarian	III	2.6
P07	41	M	Lung	I	2.2
P08	64	F	Lung	II	2.4
P09	36	F	Breast	I	0.5
P10	66	F	Gastric	III	1.6
P11	70	M	Gastric	III	3.2
P12	49	F	Cervical	I	1.8
P13	52	M	Lung	II	2.9
P14	52	F	Breast	I	2.2
P15	36	M	Gastric	II	1.3
P16	62	F	Gastric	I	0.9
P17	57	F	Colorectal	I	2.4
P18	34	M	Colorectal	I	2.8
P19	43	M	Prostate	II	1.5
P20	61	M	Lung	II	3.9
P21	60	F	Lung	II	3.4
P22	69	M	Esophageal	III	2.8
P23	61	F	Breast	II	1.8
P24	43	M	Lung	I	1.9
P25	42	M	Lung	II	1.7

F, Female; M, Male.

### 3.2 Overview of themes

Analysis identified four core themes and ten sub-themes (Table 2). These themes describe complementary aspects of participants' experiences and together map onto the principal constructs of the HBM.

**Table 2:** Summary of themes and sub-themes.

Theme	Sub-theme	Sample codes
**1. Seeking control in an uncertain world**	1.1 Reclaiming agency: an active form of self-care	• Self-initiated action • Active participation in recovery • Personal responsibility for health • Resistance to passive patient role
	1.2 Hope and belief: supplements as psychological anchors	• Doing everything possible • Emotional reassurance from routine • Symbolic meaning of supplements • Fighting spirit maintained
**2. Navigating a fragmented information landscape**	2.1 The silence of clinicians: absence of professional guidance	• Questions suppressed in consultations • Topic avoidance by clinicians • Time pressure as barrier • Unmet information need
	2.2 Peer networks and media: the dominance of informal channels	• Peer recommendation • Credibility of shared lived experience • Family-driven supplement acquisition • Social media patient groups
	2.3 Online information: navigating between fact and fear	• Information overload • Conflicting evidence online • Difficulty assessing credibility • Anxiety generated by searching
**3. The body as a battleground: supplements as a tool for resilience**	3.1 Reducing the burden of treatment: managing side effects	• Targeted symptom relief • Nausea and fatigue management • Pragmatic trial-and-error • Personal symptom toolkit
	3.2 Building reserves: strengthening the body to withstand treatment	• Immune fortification belief • Treatment tolerance as goal • Complementary logic (attack + protect) • Investment in physical reserves
**4. Silent conversations: disclosure, distrust, and the patient–provider relationship**	4.1 Fear of judgement and dismissal: choosing silence	• Anticipated dismissal by clinician • Preserving therapeutic relationship • Deference to clinical authority • Self-protective non-disclosure
	4.2 The undisclosed regimen: parallel treatment in secret	• Dual treatment regimen • Self-managed interaction risk • Hidden supplement list • Safety concern without outlet
	4.3 The desire for partnership: seeking open and empathic communication	• Desire for non-judgmental guidance • Collaborative risk–benefit review • Clinician as trusted ally • Safe communication environment

### 3.3 Theme 1: seeking control in an uncertain world

This theme captures the psychological motivation underlying much of participants' supplement use. Facing the disruption of a cancer diagnosis and the unpredictability of treatment, patients described supplements as a means of reclaiming agency and actively participating in their recovery.

#### 3.3.1 Sub-theme 1.1: reclaiming agency: an active form of self-care

Participants widely described cancer treatment as a passive experience: treatment protocols were set by clinicians, hospital schedules imposed external structure, and the disease itself was largely beyond their control. Against this backdrop, choosing and taking supplements represented one of the few domains where patients felt they could act independently. This sense of agency reduced feelings of helplessness and shifted their self-perception from passive patient to active participant in recovery.

P01 (woman, 45, breast cancer, stage II): “*From the day I was diagnosed, everything seemed to spiral out of my hands. The chemotherapy regimen was the doctor's decision; the surgery date was set by the hospital. But every morning, when I opened those bottles and took my vitamins and fish oil according to the list I had researched myself, I felt I was at least doing something for my own body. In that moment I was not a patient – I was a fighter.”*

P05 (man, 71, prostate cancer, stage III): “*The doctors handle the drugs and the radiotherapy, but they don't manage how you eat or live. I couldn't put all my hope in the hospital. I looked things up myself, took lycopene and vitamin D, and felt I was helping my body rather than just waiting to be rescued. It was about personal responsibility.”*

#### 3.3.2 Sub-theme 1.2: hope and belief: supplements as psychological anchors

For many participants, supplements carried meaning beyond their physical properties. They represented a commitment to doing everything possible to maximize the chance of recovery, and provided psychological comfort when conventional medicine offered limited reassurance. Participants described believing that supplements would help the body repair itself, or at least offset some of the toxicity of treatments such as chemotherapy, offering a sense of active hope that sustained them through difficult periods.

P02 (man, 68, lung cancer, stage IV): “*Honestly, at my stage the doctors said options were limited. But I couldn't just give up. My daughter bought mushroom extract from abroad, something said to support immunity. I don't know for certain whether it works, but taking it every day gave me a feeling of hope, a sense that I was still fighting. That feeling mattered more than almost anything else.”*

P06 (woman, 58, ovarian cancer, stage III): “*Chemotherapy takes so much out of you—you feel hollowed out. I took protein powder and multivitamins because I wanted to give my body ammunition, energy to fight the cancer cells and to repair the damage. It was a matter of faith. I believed my body needed that support.”*

### 3.4 Theme 2: Navigating a fragmented information landscape

This theme describes the complex, often frustrating process participants experienced when seeking information about supplements. A clear hierarchy of trust emerged, alongside a pervasive sense of disconnection from formal healthcare.

#### 3.4.1 Sub-theme 2.1: the silence of clinicians: absence of professional guidance

Nearly all participants reported receiving little or no proactive guidance about nutritional supplements from their oncology teams. What they encountered ranged from the topic being ignored entirely to brief, non-specific comments such as “just eat normally” or “avoid anything extra.” This professional silence created an information vacuum that pushed patients toward other sources.

P04 (woman, 39, cervical cancer, stage II): “*I wanted to ask my doctor at every appointment whether it was safe to take coenzyme Q10—I had read it was good for the heart. But he always seemed so busy, there was always a queue of patients behind me, and I swallowed the question. I had a feeling he would think I was being foolish, or would just say” useless. “They focus on your tumor markers, not on what you are taking.”*

P03 (man, 52, colorectal cancer, stage III): “*My doctor is technically excellent, but I felt he had no interest in nutrition at all. I asked if I needed to supplement anything, and he said:” Eat properly, don't take random things. “But what does” random “mean? He never explained. It felt like being left to feel your way in the dark while the one person who should have switched on the light closed the door.”*

#### 3.4.2 Sub-theme 2.2: peer networks and media: the dominance of informal channels

In the absence of professional guidance, patients turned to informal networks. Recommendations from family members, friends, and particularly other patients with cancer or cancer survivors carried strong credibility. First-hand accounts of symptom relief or improved test results were often more persuasive than any written evidence.

P01 (woman, 45, breast cancer, stage II): “*We have a WeChat group for women with breast cancer. People share everything—who noticed fewer side effects after taking something, who saw their markers improve. A lot of what I take now, including reishi spore powder and vitamin D3, came from recommendations in that group. Those women have lived this experience; I trust them more than generic expert advice online.”*

Television health programmes and web-based articles also served as common sources.

P07 (man, 41, lung cancer, stage I): “*My wife saw a health programme on TV about something anti-cancer, so she went and bought it. The children sent things home after reading articles online. Everyone meant well. I didn't always know whether it would help, but feeling cared for by the people around you – that has its own value.”*

#### 3.4.3 Sub-theme 2.3: online information: navigating between fact and fear

The internet was participants' most accessible source of information, but it generated as much anxiety as reassurance. The sheer volume of material, its contradictory nature, and the difficulty of evaluating credibility left many feeling overwhelmed.

P08 (woman, 64, lung cancer, stage II): “*My son spent every evening searching online for me. 1 day we would read that a certain supplement combined with targeted therapy might enhance the effect, and we would be excited; the next day we would find a different article saying it increased resistance, and we would be terrified. There was so much information that you could not tell what was true. Every day felt like a rollercoaster.”*

Participants described spending considerable time sifting through commercial promotions, anecdotal reports, and conflicting study summaries. The process was burdensome and frequently left them acting on incomplete judgements.

P04 (woman, 39, cervical cancer, stage II): “*Searching ”cancer nutrition“ returns thousands of results. There are sellers, personal stories, alarming headlines. I tried to read medical abstracts but they were too technical. In the end I went on gut feeling – which source looked more credible, which explanation seemed more scientific. Inside I was very uncertain.”*

### 3.5 Theme 3: the body as a battleground: supplements as a tool for resilience

This theme reflects the pragmatic dimension of supplement use. Participants described supplements as practical tools for addressing specific physical challenges imposed by cancer and its treatment, framing the body as terrain to be defended and strengthened.

#### 3.5.1 Sub-theme 3.1: reducing the burden of treatment: managing side effects

For many participants, the immediate goal of supplement use was to manage debilitating treatment-related symptoms, including nausea, fatigue, mucositis, and immunosuppression. Supplements were chosen with a specific symptom in mind, functioning as a personal toolkit for getting through the most difficult days.

P03 (man, 52, colorectal cancer, stage III): “*After chemotherapy my white cell count dropped badly—the doctors warned me about infection. A fellow patient suggested a particular mushroom broth to help bring it back up, so I made it every day. And the nausea after infusions was terrible; ginger tea genuinely seemed to ease it a little. These things were like my own small medicine chest, helping me through the worst days.”*

P06 (woman, 58, ovarian cancer, stage III): “*My biggest problem was the fatigue after chemotherapy – I could barely get out of bed. I started taking glutamine and l-carnitine and felt my energy return slightly faster. The doctor hadn't recommended them, but if something made me feel better and gave me the strength to get to the bathroom on my own, it was worth it to me.”*

#### 3.5.2 Sub-theme 3.2: building reserves: strengthening the body to withstand treatment

Beyond symptom management, participants described a strategic logic of fortification: maintaining or improving physical condition so that the body could tolerate treatment and recover from it. Many drew on a framework in which conventional treatment attacked the cancer while supplements supported the body's own defenses, viewing the two approaches as complementary rather than competing.

P01 (woman, 45, breast cancer, stage II): “*Chemotherapy is like dropping a bomb inside you—it destroys good cells along with bad ones. I took protein powder and sea cucumber extract to protect my healthy cells, to nourish them so they could rebuild. Your body is the foundation of everything; if you keep the foundation strong you can endure round after round of treatment.”*

P05 (man, 71, prostate cancer, stage III): “*My thinking was straightforward: the hospital treatment attacks the cancer; what I take myself supports my own resistance. The two work together. I can't rely only on the doctors—I have to do my part as well.”*

### 3.6 Theme 4: silent conversations: disclosure, distrust, and the patient–provider relationship

This theme addresses the communication dynamics, or more commonly the absence of them, between patients and their clinical teams regarding supplement use. Non-disclosure was common and was underpinned by fear, distrust, and the desire to preserve a fragile therapeutic relationship.

#### 3.6.1 Sub-theme 4.1: fear of judgement and dismissal: choosing silence

Many participants admitted deliberately not telling their oncologists about their supplement use. They feared being judged, criticized, or dismissed, and during a period already marked by vulnerability and stress, they were reluctant to risk any friction in their relationship with the clinical team. Silence became a form of self-protection.

P04 (woman, 39, cervical cancer, stage II): “*I did not dare tell my doctor. I was afraid he would think I didn't trust him, that I was going behind his back. He is the authority; I didn't want him to feel challenged. And what if he became less engaged with my treatment because of it? Better to say nothing.”*

P07 (man, 41, lung cancer, stage I): “*I take some traditional herbal supplements quietly. I know Western medicine might not approve. If I told my doctor, he might laugh at me or criticize me sharply. I am not young; I don't want to be lectured. It is my choice and my responsibility.”*

#### 3.6.2 Sub-theme 4.2: the undisclosed regimen: parallel treatment in secret

Non-disclosure produced a concrete patient safety risk: patients were effectively running two treatment regimens simultaneously, one documented and known to their clinical team, the other self-managed and entirely invisible to them. Some participants were aware of the potential for interactions and attempted to manage this themselves, which added another layer of burden and uncertainty.

P03 (man, 52, colorectal cancer, stage III): “*I am aware that some supplements could interact with my chemotherapy drugs; I looked this up myself. So I am careful—I stop everything for a few days before and after infusions. But that is my own judgement; the doctors know nothing about it. Sometimes I worry I might get something wrong. But I don't know who to ask.”*

P01 (woman, 45, breast cancer, stage II): “*My supplement list is longer than my prescription list. I take more than ten things each day and I keep a spreadsheet to track timing. It's like a secret project running alongside my official treatment. I feel like I'm operating in two parallel worlds.”*

#### 3.6.3 Sub-theme 4.3: the desire for partnership: seeking open and empathic communication

Beneath the non-disclosure lay a strong, largely unfulfilled desire for open dialogue with clinical professionals. Participants did not want to be told what they could not do; they wanted a trustworthy, knowledgeable partner who could help them evaluate options without judgement.

P06 (woman, 58, ovarian cancer, stage III): “*What I would love most is to hand the doctor a list of what I want to take and have him say, patiently: this one is fine, this other one might interact with your medication—hold off for now. I am not asking him to agree with everything I think. I just need a professional I can trust to help me analyse the risks and benefits. I need an ally, not a judge.”*

P22 (man, 69, esophageal cancer, stage III): “*If a nutritionist or doctor were willing to sit with me for half an hour, listen to my thinking, and then offer professional feedback, that would be wonderful. We are not being irrational—we are frightened and want to grasp every possible lifeline. If someone would tell us which lifelines are sturdy and which are dangerous, we would be deeply grateful.”*

## 4 Discussion

The findings align closely with HBM constructs. A cancer diagnosis functions as a powerful cue to action, substantially raising patients' perceived susceptibility to disease progression and perceived severity of treatment toxicity (14, 15). These heightened threat perceptions motivate the search for protective actions. The perceived benefits of supplement use reported here were multi-dimensional: psychological benefits including a sense of control and hope (Theme 1), and physical benefits in the form of symptom relief and enhanced physical resilience (Theme 3). These benefits were immediate and experiential, closely tied to patients' daily reality during treatment (13).

The principal perceived barrier was the communication environment with clinicians (Theme 2 and 4). Fear of judgement, anticipated dismissal, and time constraints in consultations all reduced the likelihood of patients disclosing their supplement use. These barriers functioned precisely as the HBM predicts: where perceived barriers are high, health behavior either goes underground or is abandoned. In this case, it went underground, creating the pattern of undisclosed parallel self-management documented in Theme 4. Self-efficacy emerged from participants' confidence in their ability to research, select, and manage supplements independently, reinforced by information from peer networks and online sources.

The theme of reclaiming agency through supplement use (Theme 1) resonates with broader research on complementary and alternative medicine in oncology. Qualitative research has shown that cancer patients often engage in a deliberate search for all available options after diagnosis, motivated in part by a desire to restore agency in the face of diagnostic uncertainty (25). The need for control has similarly been identified across both qualitative and survey-based studies as a primary driver of CAM use, with patients describing a wish to do everything possible alongside conventional treatment (26). The current data extend this by situating the control-seeking dynamic explicitly within the HBM's perceived benefits construct, emphasizing its function as a psychological coping mechanism rather than a rejection of evidence-based care.

The information-seeking patterns described in Theme 2 replicate findings from studies conducted in several countries. Patients consistently report obtaining most supplement information from non-clinical sources while receiving little from their oncology teams (2, 13, 26, 27). The qualitative data here provide texture to this pattern, showing that clinicians' silence creates an information vacuum that patients fill through peer networks and online searches. Peer-sourced information, grounded in shared lived experience, often carries greater credibility than abstract scientific evidence (28). Chen and Wang have highlighted how social media and informal health networks shape health behaviors, particularly when formal clinical communication is perceived as inadequate (28).

The pragmatic use of supplements to manage treatment side effects (Theme 3) aligns with patients' primary motivations identified in quantitative surveys, including supporting immune function, improving quality of life, and reducing the burden of specific symptoms (2, 13). These benefits are concrete and immediately verifiable by the patient, which helps to explain their persistence even in the absence of robust clinical evidence.

The non-disclosure pattern documented in Theme 4 provides qualitative explanation for quantitative data showing that up to 68% of oncologists are unaware of their patients' supplement use (9). It is also consistent with evidence from the United States showing that many patients and cancer survivors do not disclose complementary and alternative medicine use to physicians, often because clinicians do not ask or patients do not believe physicians need to know (11). Non-disclosure is not rooted in bad faith; it reflects a perceived barrier in the form of anticipated negative responses from clinicians. The practical consequence, patients managing potential drug-nutrient interactions without professional input, represents a real patient safety concern (6, 8). Integrative oncology data similarly show that patients may use multiple supplements and that professional review may lead to recommendations to discontinue products because of interaction, toxicity, or excessive-dose concerns (12). Several participants showed awareness of the interaction risk and had devised personal management strategies, which, while well intentioned, are no substitute for informed clinical oversight.

The findings suggest that prevailing approaches to patient supplement use, whether ignoring the topic or issuing blanket prohibitions, are not effective. Neither addresses the core drivers of behavior identified by the HBM; they simply increase patients' perceived barriers to communication and push their self-management further from clinical view. A different clinical posture is needed. We suggest that oncology teams adopt a collaborative, harm-reduction-oriented approach. Initiating the conversation is a critical first step (29). Rather than waiting for patients to raise the issue, or responding with caution when they do, clinicians can normalize the topic. An opening such as “Many of my patients are interested in nutrition and supplements during treatment—is that something you have been thinking about?” signals openness rather than judgement and substantially lowers the perceived barrier to disclosure. This approach is consistent with principles of patient-centered care (30). Several specific practice changes are warranted. First, qualified clinical nutrition professionals, such as registered dietitians or formally trained clinical nutritionists with oncology experience, should be integrated into standard multidisciplinary teams, giving patients access to personalized, evidence-informed guidance on diet, supplement safety, and possible interactions (31). Because patients frequently obtain nutrition information from social media, patient-facing professional profiles and institutional accounts should make formal nutrition training and clinical credentials explicit, helping patients distinguish qualified professionals from self-proclaimed advisers. Second, healthcare institutions should develop accessible, evidence-based resources that translate technical scientific evidence into generally understandable language and directly address patients' most common questions about supplement use during treatment, including practical guidance on which preparations carry meaningful interaction risks. Third, clinicians and nurses should receive training in discussing complementary health practices in a non-judgmental way, building a communication environment that supports honest disclosure (30).

The study has several limitations. As with all qualitative inquiry, the findings are not statistically generalisable; the goal is analytic transferability rather than representational coverage. Reliance on retrospective self-report introduces the possibility of recall bias. The sample was drawn primarily from a specific geographic and healthcare context in China, and the cultural and institutional factors shaping patient-clinician communication in this setting may differ from those in other countries. At the same time, the overlap between our findings and studies from the United States and Europe suggests that unmet nutrition guidance, informal information seeking, and nondisclosure are not unique to China (11, 27). Education and income were not collected systematically, limiting our ability to examine how socioeconomic position may shape supplement decision-making or access to professional nutrition advice. Future studies should examine whether these themes replicate across different healthcare systems and cultural contexts. Longitudinal study designs would allow examination of how patients' supplement-related beliefs and behaviors change across the treatment trajectory. There is also a need to design and evaluate communication interventions, such as structured consultation frameworks or patient decision aids, that directly address the barriers to disclosure identified here. Qualitative research with oncologists, nurses, dietitians, and clinical nutritionists would complement the patient perspective by illuminating how clinicians understand and respond to supplement use, helping to identify the factors that sustain the communication gap on both sides.

## 5 Conclusions

This study provides a theoretically grounded account of why and how adult cancer patients use nutritional supplements during treatment. The HBM offered a productive analytical framework, and the four themes identified (seeking control, navigating information, building resilience, and managing disclosure) together describe a coherent, if risky, form of self-management. Supplement use in this sample was not irrational or antagonistic to conventional medicine; it was a considered response to unmet psychological and physical needs in the context of a serious illness. Shifting from avoidance to proactive, non-judgmental engagement with patients about nutrition and supplements is a practical and necessary step. Embedding this change within multidisciplinary oncology care, supported by qualified clinical nutrition professionals and accessible educational resources that clearly identify formal nutrition credentials, offers a pathway toward the genuine therapeutic partnership that patients in this study consistently described wanting.

## Data Availability

The original contributions presented in the study are included in the article/Supplementary material, further inquiries can be directed to the corresponding authors.

## Supplementary material

Appendix A Semi-structured interview guide.
