# Supplementary material for: “At least i am doing something”: a health belief model-guided qualitative study of nutritional supplement use among cancer patients during treatment
Source: Front Nutr. 2026 Aug 4;13:1908173. doi: 10.3389/fnut.2026.1908173 (PMC13481245; doi:10.3389/fnut.2026.1908173)
Supplement: Supplementary file 1 [file Table_1.docx]

# **Appendix A: Semi-structured Interview Guide**

The following questions were used as a guiding framework. Supplementary probes were used as appropriate, and the interviewer followed threads introduced by participants. Questions are organised by HBM construct but were not necessarily posed in this sequence.

***Opening (rapport and context)***

• Could you tell me a little about yourself and your cancer diagnosis?

• How long have you been receiving treatment, and how has it been going?

***Perceived susceptibility and severity***

• When you were first diagnosed, how did you feel about the potential risks associated with the cancer and its treatment?

• Were there particular concerns about the illness itself, recurrence, or side effects that weighed most heavily on you?

***Cues to action***

• How did you first come to think about nutritional supplements after your diagnosis?

• Was there a particular moment, conversation, or piece of information that prompted you to start?

***Perceived benefits***

• What do you hope supplements are doing for you?

• In what ways have they affected how you feel, either physically or emotionally?

***Information seeking***

• How do you find information about supplements? Where do you usually look?

• How do you decide which sources to trust?

***Perceived barriers and self-efficacy***

• Have you encountered anything that made it difficult to find or use supplements?

• How confident do you feel in your ability to choose safe and appropriate supplements?

***Patient–provider communication***

• Have you discussed your supplement use with your doctor, nurse, or any other clinician?

• If yes: how did that conversation go? If no: what has made you hesitant?

• What kind of guidance or support would you find most helpful from your clinical team regarding nutrition and supplements?

***Closing***

• Is there anything about your experience with supplements that we have not covered and that you think is important to share?

**Table S1 COREQ (COnsolidated criteria for REporting Qualitative research) Checklist**

A checklist of items that should be included in reports of qualitative research. You must report the page number in your manuscript where you consider each of the items listed in this checklist. If you have not included this information, either revise your manuscript accordingly before submitting or note N/A.

| **Topic** | **Item No.** | **Guide Questions/Description** | **Reported on Page No.** |
| --- | --- | --- | --- |
| **Domain 1: Research team**  **and reﬁexivity** | | | |
| *Personal characteristics* | | | |
| Interviewer/facilitator | 1 | Which author/s conducted the interview or focus group? | 2.3 Data collection |
| Credentials | 2 | What were the researcher’s credentials? E.g. PhD, MD | 2.3 Data collection |
| Occupation | 3 | What was their occupation at the time of the study? | 2.3 Data collection |
| Gender | 4 | Was the researcher male or female? | 2.3 Data collection |
| Experience and training | 5 | What experience or training did the researcher have? | 2.3 Data collection |
| Relationship with  participants | | | |
| Relationship established | 6 | Was a relationship established prior to study commencement? | 2.3 Data collection |
| Participant knowledge of the interviewer | 7 | What did the participants know about the researcher? e.g. personal goals, reasons for doing the research | 2.3 Data collection |
| Interviewer characteristics | 8 | What characteristics were reported about the inter viewer/facilitator? e.g. Bias, assumptions, reasons and interests in the research topic | 2.3 Data collection |
| Domain 2: Study design | | | |
| Theoretical framework | | | |
| Methodological orientation and Theory | 9 | What methodological orientation was stated to underpin the study? e.g. grounded theory, discourse analysis, ethnography, phenomenology,  content analysis | 2.2 Sampling and recruitment |
| Participant selection | | | |
| Sampling | 10 | How were participants selected? e.g. purposive, convenience, consecutive, snowball | 2.2 Sampling and recruitment  2.3 Data collection |
| Method of approach | 11 | How were participants approached? e.g. face-to-face, telephone, mail, email | 2.2 Sampling and recruitment  2.3 Data collection |
| Sample size | 12 | How many participants were in the study? | 2.2 Sampling and recruitment  2.3 Data collection |
| Non-participation | 13 | How many people refused to participate or dropped out? Reasons? | 2.2 Sampling and recruitment |
| Setting | | | |
| Setting of data collection | 14 | Where was the data collected? e.g. home, clinic, workplace | 2.2 Sampling and recruitment  2.3 Data collection |
| Presence of non- participants | 15 | Was anyone else present besides the participants and researchers? | 2.2 Sampling and recruitment  2.3 Data collection |
| Description of sample | 16 | What are the important characteristics of the sample? e.g. demographic data, date | 3.1 Characteristics of participants |
| Data collection | | | |
| Interview guide | 17 | Were questions, prompts, guides provided by the authors? Was it pilot tested? | 2.3 Data collection |
| Repeat interviews | 18 | Were repeat inter views carried out? If yes, how many? | 2.3 Data collection |
| Audio/visual recording | 19 | Did the research use audio or visual recording to collect the data? | 2.3 Data collection |
| Field notes | 20 | Were ﬁeld notes made during and/or after the inter view or focus group? | 2.3 Data collection |
| Duration | 21 | What was the duration of the inter views or focus group? | 2.3 Data collection |
| Data saturation | 22 | Was data saturation discussed? | 2.3 Data collection |
| Transcripts returned | 23 | Were transcripts returned to participants for comment and/or correction? | 2.3 Data collection |
| Domain 3: analysis and  ﬁndings | | | |
| Data analysis | | | |
| Number of data coders | 24 | How many data coders coded the data? | 2.4 Data analysis |
| Description of the coding tree | 25 | Did authors provide a description of the coding tree? | 3.2 Overview of themes |
| Derivation of themes | 26 | Were themes identiﬁed in advance or derived from the data? | 3.2 Overview of themes |
| Software | 27 | What software, if applicable, was used to manage the data? | 2.4 Data analysis |
| Participant checking | 28 | Did participants provide feedback on the ﬁndings? | 2.4 Data analysis |
| Reporting | | | |
| Quotations presented | 29 | Were participant quotations presented to illustrate the themes/ﬁndings? Was each quotation identiﬁed? e.g. participant number | 3.2 Overview of themes  3.3 -3.6 Theme 1 -4  TABLE 2 Summary of themes and sub-themes |
| Data and ﬁndings consistent | 30 | Was there consistency between the data presented and the ﬁndings? | 3.3 -3.6 Theme 1 -4  TABLE 2 Summary of themes and sub-themes |
| Clarity of major themes | 31 | Were major themes clearly presented in the ﬁndings? | 3.3 -3.6 Theme 1-4  TABLE 2 Summary of themes and sub-themes |
| Clarity of minor themes | 32 | Is there a description of diverse cases or discussion of minor themes? | 4 Discussion |

Developed from: Tong A, Sainsbury P, Craig J. Consolidated criteria for reporting qualitative research (COREQ): a 32-item checklist for interviews and focus groups. *International Journal for Quality in Health Care*. 2007. Volume 19, Number 6: pp. 349 – 357

**Once you have completed this checklist, please save a copy and upload it as part of your submission. DO NOT include this checklist as part of the main manuscript document. It must be uploaded as a separate file.**
